# Governing Ethical Tensions in Youth Digital Mental Health Research

**DOI:** 10.2196/88622

**Published:** 2026-06-30

**Authors:** Kristin Berre Ørjasæter, Stefan Rennick-Egglestone, Kristin Øksendal Børresen, Cathrine Fredriksen Moe, Anna Leiler, Helga DI Sigurdardóttir, Victor Manuel Pèrez Colado, Malle Vogelsang, Teddy Nambazira, Jone Trovåg, Victor Valderaune, Ingunn Skjesol, Fiona Ng, Ottar Ness, Mike Slade

**Affiliations:** 1 Division of Health and Community Participation, Faculty of Nursing and Health Sciences, Nord University, Finn Christiansgt 1, Namsos, Trøndelag, 7801, Norway, 47 74212337; 2 Recovery Research Team, Institute of Mental Health, University of Nottingham, Nottingham, United Kingdom; 3 Institute of Mental Health, Nottingham Biomedical Research Centre, Nottingham, United Kingdom; 4 Division Health and Community Participation, Faculty of Nursing and Health Science, Nord University, Bodø, Nordland, Norway; 5 Division of Health and Community Participation, Faculty of Nursing and Health Sciences, Nord University, Levanger, Trøndelag, Norway; 6 Division of Games and Entertainment Technology, Faculty of Social Sciences, Nord University, Levanger, Trøndelag, Norway; 7 Mental Health Youth Organization, Oslo, Norway; 8 Division Health and Welfare, Åfjord Municipality, Åfjord, Norway; 9 Department of Education and Lifelong Learning, Faculty of Social and Educational Sciences, Norwegian University of Science and Technology, Trondheim, Norway

**Keywords:** cocreation, consent, data governance, digital ethics, digital health, participatory research, recovery narratives, research ethics, youth, innovation

## Abstract

As mental health research increasingly aims to generate societal impact, researchers operate at the intersection of innovation and ethical responsibility. Drawing on experiences from the cocreated NEON Young Norway Study on youth recovery narratives, this viewpoint identifies four ethical tensions that arise from the existing governance frameworks in youth digital mental health research: (1) balancing safeguarding against harm with youth participation, (2) protecting privacy without undermining authentic storytelling, (3) governing unpredictable outcomes of cocreated research, and (4) meeting ethical and legal standards while ensuring youth-friendly communication. These tensions highlight limitations in mental health research that adopts participatory and digital approaches, as this often struggles to accommodate iterative designs, narrative data, and cross-sector collaboration. We argue that responsible youth mental health research requires ethics to be understood as a dynamic, participatory practice that supports safe and equitable inclusion, rather than having a focus on risk prevention. Ethical governance, therefore, needs to evolve toward proportionate, context-sensitive approaches that can enable innovation while protecting young people’s rights, agency, and voices.

## Background

Youth mental health is a global public health priority, yet service provision continues to fail to meet the need [1]. In response, digital health technologies have become increasingly prominent in mental health research and practice, a trend further accelerated by the COVID-19 pandemic [2]. At the same time, first-person mental health recovery narratives (RNs) are increasingly being recognized as valuable resources in mental health support [3], with potential benefits for recovery processes [4,5]. Together, these developments have motivated research that integrates digital innovation with narrative and cocreated approaches in youth mental health.

Concurrently, major funding bodies, including Horizon Europe [6], the European Research Council [7], and the Norwegian Research Council [8], increasingly prioritize research that combines technological innovation with user involvement, cocreation, and real-world impact. This aligns with a broader shift in contemporary research toward cross-sectoral collaboration, social accountability, and responsiveness to complex societal challenges [9]. However, such approaches can strain established arrangements for research governance [10,11]. Ethics regulators operate under mandates designed to safeguard participant welfare, ensure data protection, and uphold procedural compliance, thereby producing responsive and respectful research [10]. These rules can generate practical tensions for researchers, particularly in projects involving rapidly evolving technologies or novel data practices. Balancing timely innovation with robust ethical oversight has become an ongoing challenge [12,13].

Established global research ethics frameworks aim to safeguard participants’ rights and welfare [14-17], and they are complemented in Norway by field-specific guidelines [18-21] and legal instruments [22-24] governing medical, health, social science, and digital research. Nevertheless, digital mental health care highlights the need to embed ethical considerations throughout the research process [13], particularly in studies introducing novel digital or participatory approaches that challenge traditional norms [25]. Previous research also demonstrates variation in the articulation and management of ethical issues, including approvals and informed consent [12].

When research involves young people, ethical considerations are further amplified, demanding sustained attention to consent, confidentiality, and risk mitigation across all stages of the research lifecycle [26-30]. Trust is particularly central in youth mental health research, where there is an emphasis on close collaboration with end users and industry partners, as well as the incorporation of trust-building elements, such as accessible privacy policies and clear visual explanations of complex privacy terms [31].

In this study, ethical tensions are understood as situations in which ethical principles, governance requirements, or stakeholder expectations generate uncertainty about responsible practice. This paper aims to examine such tensions in cocreated digital youth mental health research. Drawing on empirical experiences from the NEON Young Norway Study, which integrates RNs and digital technologies, it reflects on the implications for ethical governance.

## The NEON Young Norway Study

The NEON Young Norway Study: Facilitating Narrative Experiences Online to Provide Mental Health Support for Young People (2023‐2027) is a 4-year interdisciplinary research project funded by the Norwegian Research Council. It involves young people aged 16 to 30 years who contribute RNs in various formats (text, audio, video, and images), both newly authored and previously published narratives, with explicit consent for reuse. These materials are used as digital resources in games, interactive media, learning materials, and a prototype low-threshold digital intervention offering tailored support based on user preferences. Tailoring is achieved through manual narrative tagging and user-directed choice, enabling young people to select stories by format, thematic content, and/or emotional tone.

The project comprises 4 work packages (WPs): WP1 analyzes RNs and explores the rationale for sharing; WP2 examines how young people, their peers, and practitioners understand, experience, and use RNs; WP3 uses RNs donated by young people to guide the development of games and interactive media; and WP4 develops and tests prototype interventions to assess feasibility and inform potential commercialization.

The study is co-developed with young people, clinical partners, and technology developers and involves cocreation. We define cocreation as a collaborative process in which researchers, stakeholders, and end users jointly generate research knowledge [32], with the aim of producing actionable knowledge grounded in the perspectives and priorities of those most affected by the research. The NEON Young Norway Study is also supported by 2 expert boards: the Project Advisory Board, comprising researchers in mental health recovery, digital health technology, and youth involvement, and the Lived Experience Advisory Panel (LEAP), consisting of young individuals, age 16 to 30 years, with various backgrounds and experiences with mental health challenges and recovery. Both boards actively shape the research design, analysis, and development of youth-centered digital tools.

This paper explores the ethical tensions encountered by the NEON Young Norway Study, particularly regarding interactions with Norwegian regulatory bodies.

The study collects large volumes of sensitive data, which are processed and stored in Services for Sensitive Data, a secure platform designed for handling sensitive health-related research data. Access is strictly regulated according to the General Data Protection Regulation (GDPR) principles of data minimization and integrity and confidentiality (GDPR Article 5(1)(c) and (f)), and only project members with a documented need-to-know can access identifiable information. As the study processes special category data, all activities follow the research exemption (GDPR Article 9(2)(j)) and the relevant Norwegian Health Research Act (Sections 4, 17, and 33).

## Analytical Approach

### Project Materials and Analytical Process

This paper draws on project documentation from the NEON Young Norway Study (October 2023–April 2026), including meeting notes, correspondence, ethical approval materials, reflexive team discussions, and input from both the Project Advisory Board and the LEAP.

We conducted a structured document analysis, inspired by the work of Bowen [33], combined with reflexive thematic analysis as outlined by Braun and Clarke [34]. This approach conceptualizes meaning as something that is coconstructed through analytical engagement and requires explicit attention to researcher positionality and assumptions.

The first author led the analysis by organizing and reviewing all the project materials related to ethical issues, generating initial codes, and developing themes. Theme development and refinement occurred through iterative discussions with the coauthors in individual and small-group meetings, functioning as critical dialogue rather than consensus coding, consistent with reflexive thematic analysis’s epistemological stance. Through repeated engagement with the material, 4 overarching ethical tensions were identified.

This analysis does not aim to provide an exhaustive account of all ethical discussions within the project. Rather, it seeks to illuminate the key ethical tensions that have emerged in practice and that appear relevant to youth mental health research involving digital innovation. The material analyzed consists of project documentation created as part of the project rather than systematically generated research data, consistent with participatory and reflexive methodological approaches in which insights are expected to emerge from engagement with practice, and collective interpretation.

### Ethical Approval Process for the NEON Young Norway Study

In Norway, the ethical approval process for medical and health research includes the assessment of both ethical considerations and data processing arrangements under the GDPR, with data protection compliance supported by the Norwegian Agency for Shared Services in Education and Research (Sikt) through guidance and secure data services. Within this governance framework, the NEON Young Norway Study underwent an extensive ethical approval process because of its innovative design, cocreated approach, and handling of sensitive data. The initial application proposed consent as the legal basis for data processing and included the potential future use of RNs in digital intervention. It included provisions for both research and possible commercial use from project inception, used standardized participant information sheets based on the Sikt template, and allowed participant discretion in personal disclosures in their RNs, while advising the minimal inclusion of third-party data. The approval process spanned approximately 9 months and involved multiple revisions.

Final ethical approvals were granted by the Regional Committees for Medical and Health Research Ethics (REC) (May 2024, REC/664616) and the Sámi Expert Ethical Committee for Sámi Health Research, which provided Collective Sámi Consent (February 2024, SEKSH/1119960). Additionally, Sikt assessed compliance with the data protection regulations (May 2024, Sikt/545210).

Key changes during the approval process included the completion of a Data Protection Impact Assessment and a shift in the GDPR legal basis from consent to public interest (GDPR Articles 6(1)(e) and 9(2)(j)). This reflected recognition that cocreated, publicly funded youth mental health research is more appropriately governed through institutional responsibility and safeguards than through individual legal consent alone. Participants retain the right to withdraw from the study; however, withdrawal does not automatically require the deletion of data already included in completed analyses, publications, or derived materials. Identifiable personal data are normally deleted upon withdrawal, while materials already produced remain unchanged in line with public interest and research integrity requirements. Further revisions clarified anonymization and pseudonymization practices, adapted participant materials to communicate risks more clearly and limit consent to the initial research phase, and replaced Sikt templates with those required by REC.

The approval process is treated as an analytic site through which ethical tensions are identified and examined, informing reflections on governance challenges in cocreated digital youth mental health research.

## Ethical Tensions in Youth Digital Mental Health Research

### Overview

Based on our experience in obtaining ethical approval for the NEON Young Norway Study, we identified 4 key tensions in designing and conducting youth mental health research. While these tensions are analytically distinct, they are interconnected and unfold within broader governance conditions shaped by cocreation, digital infrastructures, and uncertainty over future data use. Emerging within a Norwegian research governance context characterized by a highly coordinated ethics infrastructure and strong data protection support, these tensions illustrate how ethical dilemmas are shaped and negotiated in practice. We present the tensions below, moving from widely shared ethical concerns in youth research to more specific governance challenges arising from digital, narrative-based, and cocreated research practice.

### Tension 1: Balancing Safeguarding Against Harm With Youth Participation

Health research relies on robust protection mechanisms, particularly when the participants are categorized as vulnerable [14,35]. However, extensive harm-prevention measures may constrain autonomy and limit opportunities for research engagement. This tension centers on balancing the ethical responsibility to minimize harm with the imperative to support youth participation in mental health research.

Young people with lived experience of mental health challenges are frequently positioned within 2 vulnerability categories, as minors and as individuals experiencing mental ill health. Consequently, safeguarding responsibilities are often delegated to adult gatekeepers, such as parents, teachers, or professionals, who regulate access to research participation [28,36]. While vulnerability requires careful recruitment and informed participation [18], group-level vulnerability framings may intensify safeguarding practices to the point of overprotection and exclusion from research [37]. Such protectionist approaches, though well intentioned [11], risk being disempowering when decisions are made on behalf of young people rather than with them [28]. Scholars have therefore questioned who defines acceptable risk [11] and have argued for the greater recognition of minors’ autonomy, emphasizing that young people should be respected, regardless of their carers’ preferences [38], and treated as competent social actors [29].

These debates were evident in the NEON Young Norway Study, in which the inclusion of 16‐18-year-olds with mental health conditions triggered sustained deliberations about parental consent and additional safeguarding measures, despite Section 17(b) of the Norwegian Health Research Act granting consent competence from the age of 16 years [24]. These discussions unfolded through ongoing written correspondence and digital meetings between the research team and the external data governance actors responsible for data protection oversight. Although parental consent was not legally required for these individuals, some regulators would recommend it as an additional precaution. However, the project chose to respect the young people’s legal and moral autonomy while implementing multiple support structures, including clear information about the potential emotional burdens and benefits of participation, access to low-threshold support services, and procedural safeguards such as researcher contact in cases of distress and systematic screening of submissions for indicators of acute risk. In line with Potthoff et al [36], these measures illustrate how safeguards can be implemented without adopting overly risk-averse approaches that unnecessarily limit youth participation in mental health research. Overall, this tension illustrates that safeguarding young people in mental health research is not solely about reducing risk but is also about creating safe conditions that actively support participation and autonomy.

### Tension 2: Protecting Privacy Without Undermining Authentic Storytelling

Data protection regulations and research ethics frameworks require anonymization or pseudonymization to protect participants from identification and potential harm, particularly for groups often categorized as vulnerable [16,18,22,24]. However, this deidentification may compromise narrative integrity by removing contextual elements that are essential to meaning and authenticity. This creates an ethical tension between maintaining robust confidentiality and preserving authentic representation, ownership, and recognition.

In qualitative research, deidentification typically involves modifying names, locations, or contextual details to protect participants. These practices affect how narratives are interpreted and experienced, potentially weakening voice and relational meaning [39,40]. This concern is salient in mental health research relating to lived experience narratives. Because these narratives derive meaning from contextual details, such as names, places, relationships, and voice, pseudonymization may reduce narrative agency, ownership, and social impact, particularly for individuals living with mental health challenges [39]. Rennick-Egglestone et al [39] further argue that altering narratives in this way may unintentionally reinforce historical silencing and discrimination, thereby undermining the empowering potential of narrative accounts.

These dilemmas became concrete in the NEON Young Norway Study: tensions emerged between the institutional interpretation of the GDPR and the commitments to narrative ethics in practice. Young people contributed RNs, some of them previously published and others created specifically for the project. Institutional interpretations of the GDPR [16] and the Norwegian data protection legislation [22] argue for the restriction of the use of personal identifiers in unpublished narratives, generally limiting disclosure to age and first name or a participant-chosen pseudonym. While allowing the participants to select their own pseudonyms aligns with Morrow’s [27] approach, this practice also constrained their ability to claim authorship and raised concerns about ownership and recognition, as noted by other scholars [11]. For the previously published narratives, identifiers were retained, but the data protection experts required explicit communication of the potential risks related to recognition. These requirements prompted sustained ethical deliberation within the research team about how far narratives could be edited to meet pseudonymization standards without eroding authenticity, relational meaning, and the young people’s ability to represent their experiences on their own terms. At the same time, decisions about retaining contextual detail had to balance respect for participant agency against responsibilities to protect privacy and third-party interests. Together, these considerations illustrate that efforts to safeguard privacy may unintentionally diminish narrative integrity, authorship, and the very voices such research seeks to amplify.

### Tension 3: Governance for the Unpredictable Outcomes of Cocreated Research

Cocreated research involves iterative development, emergent aims, and evolving data use, generating ethical uncertainty for governance systems built around stable purposes, predefined data pathways, and one-time informed consent. Consequently, transparency becomes difficult to achieve in advance, as future aims and data uses cannot be fully specified [41]. This has led to growing attention to dynamic, process-oriented consent approaches that frame consent as an ongoing ethical practice rather than a single procedural decision [41]. The central tension is how to govern unpredictable and evolving research outcomes while maintaining participant autonomy and accountability.

This tension is intensified in youth mental health research that uses cocreative and digital approaches. Research involving young people and sensitive topics entails heightened expectations regarding clear information about the purpose, procedures, and implications [14,18]. At the same time, cocreative research relies on iterative and adaptive processes [11], which may alter both research aims and future data uses. Digital mental health interventions further extend the temporal horizon of potential data use, introducing uncertainties related to long-term implications and the reuse and transformation of narrative materials. As a result, ethical expectations of predictability and control can come into tension with research approaches that rely on emergence, flexibility, and cocreation.

Drawing on our analysis of the interactions with research ethics bodies and data governance stakeholders, uncertainty in the NEON Young Norway Study concerned both immediate research use and the potential for unpredictable future outcomes, including evolving data pathways for participants’ RNs. The study explored young people’s understandings and use of RNs, while cocreating and testing a digital intervention prototype based on these narratives. Although the participants could consent to the use of their narratives across multiple research WPs, it was not possible to specify the final form of the intervention or the longer-term outcomes and implications of using the RNs. If the prototype proved feasible beyond the research phase, additional data pathways could also emerge, extending uncertainty beyond the initial research context. While any future digital mental health intervention with commercial potential would not involve the direct reuse of the RNs collected during the research phase, the prospect of later public or commercial deployment nevertheless shaped how future data pathways were anticipated and governed. The participants were therefore informed from the outset that they might be recontacted to consider renewed consent for new forms of use, positioning commercialization as a prospective uncertainty rather than a predetermined outcome.

These examples of uncertainty and unpredictability reflect ongoing debates in digital health ethics, where one-time consent models are widely recognized as insufficient for research involving iterative and evolving data practices [41]. Such models assume stable research purposes and clearly defined data uses, assumptions that are difficult to uphold in cocreated processes. As a result, digital mental health researchers increasingly advocate consent-forward or process-oriented approaches to consent, including dynamic consent, emphasizing ongoing, affirmative, and proactive engagement with participants, as well as agency for the participants over how data travel, transform, and are repurposed over time [42]. Nonetheless, even procedurally compliant consent cannot eliminate unpredictability in future outcomes, underscoring the need to govern uncertainty and unpredictability as inherent features of cocreated digital research rather than as an ethical failure.

### Tension 4: Meeting Ethical and Legal Standards While Ensuring Youth-Friendly Communication

This tension arises from the need to comply with ethical and legal requirements while simultaneously ensuring that study information is accessible and meaningful to young participants. Health research involving human participants typically requires prior approval from ethics regulators [14], a process that often prioritizes formal language, detailed documentation, and the use of secure digital systems for managing sensitive data. While these standards are essential for legal compliance, data protection, and transparency, they may conflict with the ethical requirement to provide clear, developmentally appropriate communication that supports equitable participation.

Youth mental health research presents particular challenges in this regard, not because young people are uniquely variable but because developmental diversity intersects with age-based consent norms and heightened ethical expectations of protection and transparency. The participants constituted a heterogeneous group with varying cognitive capacities, digital skills, and lived experiences. Empirical research shows that long, text-dense consent materials reduce comprehension and engagement among young people, who often skim or disengage from complex documents [11,28,43]. From a cognitive perspective, some individuals have a limited working-memory capacity, meaning that excessive or highly complex information undermines their understanding rather than enhancing it [44]. Digital recruitment and consent procedures may further amplify this problem through multistep workflows, nonintuitive interfaces, and cumulative cognitive load, thereby creating barriers to participation for groups already at risk of exclusion, including vulnerable or hard-to-reach populations [45]. In youth research, concerns about comprehension and cognitive overload may in turn prompt recommendations for additional gatekeeping measures, parental consent, or more restrictive inclusion criteria.

In the NEON Young Norway Study, this tension became evident through our analysis of how the participant information materials evolved during the ethics approval process. As legal and data-protection requirements were incorporated into standard templates, one information sheet expanded from approximately one and a half to almost four pages during the approval process. This directly conflicts with guidance from LEAP members, who emphasize brevity and clarity, and aligns with evidence that lengthy, text-dense materials reduce comprehension and meaningful engagement among young people. Although the project sought to mitigate these challenges through web-based information using bullet points, chunking, simplified language, and short video explanations, the technical consent solution based on the Services for Sensitive Data, with digital ID, multiple steps, and separate uploads, remained demanding for some participants. In this context, legal precision and data security were prioritized at the expense of usability and accessibility.

This tension demonstrates that ethical governance is a matter not only of regulatory compliance but also of how information is communicated. In youth mental health research, ethical practice requires consent solutions that are comprehensible, accessible, and tailored to participants’ capacities, while remaining within existing regulatory frameworks.

## Ethical Implications

### Overview

This paper has identified 4 interrelated ethical tensions shaping youth mental health research, particularly within cocreated, narrative-based, and digitally mediated approaches. Four implications of these tensions are now identified. These implications invite ongoing reflection on how ethical responsiveness can be sustained amid evolving contexts, participant agency, and methodological change.

### Implication 1: Reframing Risk Through Youth Agency and Harm Reduction

A first implication concerns the need to move beyond protectionist framings of risk that position young people primarily as vulnerable participants requiring safeguarding by adults. While safeguarding remains essential, overly protectionist models may inadvertently exclude young people, marginalize experiential knowledge [11], and undermine the ethical value of participation itself [11,28,36].

Reconceptualizing risk through a harm-reduction lens allows for a broader ethical assessment that includes not only potential emotional distress but also harm related to research exclusion, loss of agency, and the silencing of youth perspectives. Framing young people as competent social actors shifts ethical attention from risk avoidance toward ethically supported processes in which both potential burdens and potential benefits are considered. Within this framing, safeguards remain a necessary component of ethical practice, but attention also needs to be drawn to how safeguards intersect with agency, informed decision-making, and the intrinsic ethical value of participation [36]. Protectionist governance approaches can constrain participation and limit the social and epistemic value of research based on lived experience [36]. This is particularly salient in youth mental health research, where an overemphasis on risk avoidance restricts innovation and diminishes social value.

### Implication 2: Preserving Narrative Integrity While Ensuring Privacy

A second implication concerns the tension between preserving narrative integrity and meeting robust privacy requirements in youth mental health research. Narrative meaning, ownership, and authenticity are often inextricably linked to context, bringing participants’ rights to recognition and narrative agency into tension with nonnegotiable legal and ethical constraints, such as third-party privacy [35], GDPR requirements [16], and national data protection standards [22,24].

Empirical experience shows that ethical practice in this space involves navigating these constraints through context-sensitive judgment rather than treating anonymization or pseudonymization as a purely technical exercise. In practice, research teams may differentiate between raw narratives, analytically processed versions with selective pseudonymization, and publication versions requiring additional masking. However, these approaches do not resolve the underlying tension. In the NEON Young Norway Study, legal pseudonymization requirements at times conflicted with young people’s intentions regarding voice, recognition, and authorship, including the possible wish to be identifiable. Narrative alteration could reshape relational meaning and diminish agency, while extensive signaling of edits risked disrupting coherence and interpretive integrity.

These dilemmas illustrate that no procedural solution can fully reconcile privacy protection with narrative integrity. Ethical practice therefore emerges as an ongoing engagement with how editorial decisions affect voice, tone, and meaning, especially when narratives include relational dynamics, third-party references, or context essential to lived experience. When deidentification is applied rigidly, there is a risk of altering meaning or reproducing silencing, thereby undermining the ethical aims of narrative research [39,40].

When viewed in this way, preserving narrative integrity becomes not only an ethical issue but also a matter of justice [46]. Analysis of lived experience in mental health research suggests that when privacy protections are made explicit and negotiated, narratives retain a greater capacity for agency, connection, recognition, and social impact [39,46,47].

### Implication 3: Governing Uncertainty Through Proportionate, Flexible, Process-Oriented Consent

A third implication arising from these tensions is the limitation of one-time, procedural consent approaches in cocreated research in which the research aims, methods, and data pathways evolve over time. The ethical challenge associated with this tension stems not only from uncertainty about future data use, but also from the way in which this uncertainty is communicated and governed. This calls for consent to be reconceptualized not as a discrete event but as an ongoing ethical practice that is responsive to change over time [11,41].

From this perspective, process-oriented consent can be understood as supporting ethical responsiveness through proportionate and flexible engagement rather than exhaustive specification in advance. Practices such as periodic opportunities for reflection on consent, layered information provision, and selective reengagement when data use changes can support ongoing ethical practice [40]. Importantly, such practices retain ethical relevance even when the legal basis for data processing is the public interest. They also function as safeguards for transparency, communication, and respect for participant agency [48]. At the same time, the literature highlights the importance of proportionality, given the risks of increasing the burden on participants and of consent fatigue in digitally mediated research [48,49].

Consent processes are often calibrated to participants’ capacities and preferences, particularly for young individuals, who may benefit from structured, accessible, and multimodal information formats [26,43]. Regulatory templates may offer a useful starting point, yet empirical experience shows that they often require adaptation to achieve proportionality and accessibility. This highlights how reliance on age or mental health status as categorical markers of consent capacity becomes ethically problematic and brings to the foreground the importance of individualized and context-sensitive assessments rather than restrictive gatekeeping based on group-level assumptions [11].

### Implication 4: Establishing Cross-Sector Ethical Reflection Spaces

The final implication concerns the need for spaces that support ongoing, collective ethical reflection in digital youth mental health research. In contexts characterized by narrative-based innovation, digital mediation, and cocreation, ethical challenges cannot be fully anticipated or resolved through upfront review alone. Dedicated reflection spaces can instead help to embed ethics as an integrated and continuous component of research practice, strengthen the capacity of researchers and collaborators to navigate ethical tensions, and facilitate shared understanding across the sectors involved in governance, care, and technology development.

Such spaces can support proportionate and adaptive governance by grounding ethical deliberation in real-world practice, enabling mutual learning about consent, privacy, and youth participation, and fostering forms of ethical accountability that develop alongside innovation rather than in response to it. Practically, these spaces may take diverse forms, ranging from interdisciplinary ethics workshops and digital forums for sharing dilemmas to case-based training initiatives, thematic events, or scholarly occasions for reflecting on how ethical tensions are navigated in cocreated and technology-driven research.

Drawing on “ethics as practice” perspectives such as ethics maps [13], this approach conceptualizes ethics as a living and adaptive practice, one that remains responsive to new knowledge, shifting contexts, and emerging ethical challenges over time.

## Limitations

This study is situated within the single national context of Norway, which is characterized by a well-established and highly coordinated research ethics infrastructure. Norwegian research governance includes ethics guidelines for medical and health research, social sciences and humanities, and internet and technology studies; robust institutional data protection expertise; and a comprehensive infrastructure for secure data management. These conditions shape how ethical tensions arise and are addressed in our project; in contexts with other regulatory traditions, resource levels, or institutional arrangements, the conditions may be different.

A second limitation concerns transferability. While we argue that the ethical tensions discussed might be conceptually relevant in other contexts, our empirical insights are grounded in Norwegian governance mechanisms. Distinguishing between what is context-dependent and what holds broader conceptual value is, therefore, essential. Our analyses should be read as analytically suggestive, rather than universally generalizable.

Finally, our reflections are situated within a long-term, cocreated research process. Ethical tensions did not arise for us as discrete, solvable moments but as iterative, situated dilemmas requiring ongoing negotiation over time. Their salience shifted as the project evolved, and different teams, timelines, or research designs might bring alternative tensions to the surface. We therefore offer this study not as an exhaustive mapping but as an invitation to continued inquiry into how ethical tensions manifest themselves and evolve across diverse research governance landscapes.

## Concluding Remarks

As advances in digital tools, participatory approaches, and youth-driven methodologies reshape mental health research, new ethical complexities inevitably emerge. Existing frameworks offer an essential foundation, yet they do not fully address the challenges introduced by digital technologies, participatory methods, narrative data, and cross-sector collaboration. The 4 tensions identified in this viewpoint highlight areas in which ethical governance must evolve, and the accompanying implications identify how this evolution may be approached in practice.

Ethics in youth mental health research should be understood as a dynamic, iterative practice, grounded in respect for persons, beneficence, and justice, yet flexible enough to accommodate innovation. Addressing these tensions requires context-specific ethical judgment supported by critical reflection, methodological flexibility, and youth involvement in shaping ethical decisions [11] on matters ranging from information design and consent processes to risk-benefit assessments, narrative representation, and data protection.

Looking forward, the field would benefit from proportionate governance models, clearer guidance on digital and narrative consent, and empirical research examining how young people engage with layered consent, multimodal information formats, and deidentification processes. Ethical practice must keep pace with technological and methodological change, ensuring that innovation enhances, rather than constrains, youth participation, autonomy, and recognition. By adopting adaptive consent practices, a balanced approach to risk, transparent narrative stewardship, and cross-sector ethical dialogue, researchers and institutions can support both ethical rigor and authentic youth engagement.

Finally, while innovation should never override ethical and legal obligations, overly rigid compliance may hinder ethical learning and cocreation. The challenge is to strike a context-sensitive balance that protects participants’ rights and well-being without silencing their voices or constraining collaborative knowledge production. As research continues to evolve, so too must our ethical reflexivity, ensuring that integrity grows alongside scientific and technological development.

## Abbreviations

GDPR: General Data Protection Regulation
LEAP: Lived Experience Advisory Panel
REC: Regional Committees for Medical and Health Research Ethics
RN: recovery narrative
WP: work package
